# Outcomes for Dostarlimab and Real-World Treatments in Post-platinum Patients With Advanced/Recurrent Endometrial Cancer: The GARNET Trial Versus a US Electronic Health Record-Based External Control Arm

**DOI:** 10.36469/001c.77484

**Published:** 2023-09-08

**Authors:** Scott Goulden, Qin Shen, Robert L. Coleman, Cara Mathews, Matthias Hunger, Ankit Pahwa, Rene Schade

**Affiliations:** 1 GSK, London, UK; 2 GSK, Collegeville, Pennsylvania, USA; 3 Texas Oncology, The Woodlands, Texas, USA; 4 Alpert Medical School of Brown University, Providence, Rhode Island, USA; 5 ICON plc, Munich, Germany; 6 ICON plc, Bangalore, India; 7 ICON plc, Reading, UK

**Keywords:** uterine neoplasms, endometrial cancer, dostarlimab, chemotherapy, immunotherapy, treatment outcome, survival

## Abstract

**Background:** Patients with advanced or recurrent endometrial cancer (EC) have limited treatment options following platinum-based chemotherapy and poor prognosis. The single-arm, Phase I GARNET trial (NCT02715284) previously reported dostarlimab efficacy in mismatch repair–deficient/microsatellite instability–high advanced or recurrent EC.

**Objectives:** The objective of this study was to compare overall survival (OS) and describe time to treatment discontinuation (TTD) for dostarlimab (GARNET Cohort A1 safety population) with an equivalent real-world external control arm receiving non-anti-programmed death (PD)-1/PD-ligand (L)1/2 treatments (constructed using data from a nationwide electronic health record–derived de-identified database and applied GARNET eligibility criteria).

**Methods:** Propensity scores constructed from prognostic factors, identified by literature review and clinical experts, were used for inverse probability of treatment weighting (IPTW). Kaplan-Meier curves were constructed and OS/TTD was estimated (Cox regression model was used to estimate the OS-adjusted hazard ratio).

**Results:** Dostarlimab was associated with a 52% lower risk of death vs real-world treatments (hazard ratio, 0.48; 95% confidence interval [CI], 0.35-0.66). IPTW-adjusted median OS for dostarlimab (N=143) was not estimable (95% CI, 19.4–not estimable) versus 13.1 months (95% CI, 8.3-15.9) for real-world treatments (N = 185). Median TTD was 11.7 months (95% CI, 6.0-38.7) for dostarlimab and 5.3 months (95% CI, 4.1-6.0) for the real-world cohort.

**Discussion:** Consistent with previous analyses, patients treated with dostarlimab had significantly longer OS than patients in the US real-world cohort after adjusting for the lack of randomization using stabilized IPTW. Additionally, patients had a long TTD when treated with dostarlimab, suggesting a favorable tolerability profile.

**Conclusion:** Patients with advanced or recurrent EC receiving dostarlimab in GARNET had significantly lower risk of death than those receiving real-world non-anti-PD-(L)1/2 treatments.

## BACKGROUND

Endometrial cancer (EC) is the fourth most common cancer among women in developed countries, and its incidence is steadily increasing.^1,2^ The prognosis for patients with advanced or recurrent EC is poor, reflected by a 5-year survival rate of 18% for those diagnosed with distant metastatic disease.^2^ Further, treatment options have been historically limited for patients with advanced or recurrent EC whose disease progresses on or after first-line (1L) platinum-based chemotherapy (PBCT), with limited efficacy of chemotherapy in this setting and no standard of care for second-line (2L) chemotherapy.^3,4^ Response rates as low as 10% to 15% have been observed with available 2L options.^3^ There is, therefore, a clear need for access to effective novel therapies for the treatment of patients with advanced or recurrent EC in the 2L setting.

Approximately 25% of EC tumors are mismatch repair–deficient (dMMR)/microsatellite instability–high (MSI-H).^5,6^ While there is conflicting evidence on the prognostic value of MMR/MSI status for patients with EC,^7–10^ it nonetheless represents a biomarker that can inform therapeutic decision-making beyond chemotherapy-based regimens; immune checkpoint inhibitors such as antibodies against programmed death (PD)-ligand (L) 1/2 or their receptor, PD-1, are emerging as promising treatments for this population of patients.^11,12^ Examples of these include the anti-PD-1 antibodies pembrolizumab^11^ and dostarlimab.^12^

The efficacy and safety of dostarlimab in patients with dMMR/MSI-H advanced or recurrent EC was assessed in the single-arm Phase I GARNET trial (NCT02715284).^13^ Based on results from GARNET, dostarlimab became the first anti-PD-1 treatment approved in the European Union for advanced or recurrent EC,^14^ specifically as a monotherapy in adult patients with advanced or recurrent dMMR/MSI-H EC that has progressed on or after treatment with a platinum-containing regimen.^13^ In the United States (US), dostarlimab is approved as a monotherapy in adult patients with advanced or recurrent dMMR EC that has progressed on or after a platinum-containing regimen.^15^ The GARNET trial did not include a comparator arm and, as such, dostarlimab efficacy has not yet been compared with currently available treatment paradigms.

An external control arm study uses external data to create a comparator arm for clinical evaluation and applies statistical methods to evaluate the effectiveness of an intervention against the external control.^16^ Such studies are useful in the context of patient populations that are challenging to recruit or assess in randomized clinical trials.^16^ The lack of random assignment of patients can be mitigated by applying well-defined inclusion and exclusion criteria to select individual patients from a data source, and statistically balancing baseline characteristics between the intervention and external cohorts.^17^ The validity of external control arms, such as those derived from real-world electronic health records (EHRs), has been recognized by regulatory and health technology assessment (HTA) authorities,^18,19^ and evidence from such studies has successfully been used to support drug regulatory applications and HTA submissions.20

The primary objective of this study was to compare overall survival (OS) of patients with dMMR/MSI-H advanced or recurrent EC treated with dostarlimab in the GARNET trial versus real-world patients with advanced or recurrent EC receiving current treatment paradigms. To achieve this, an external control arm of real-world patients was built using the Flatiron Health database, which contains real-world individual patient data from the US. In addition to OS, the time to treatment discontinuation (TTD) was assessed as an exploratory objective.

## METHODS

### Study Design

GARNET is an ongoing multicenter, single-arm, open-label Phase I clinical trial of dostarlimab monotherapy in adult patients with advanced solid tumors. Cohort A1 of Part 2B of the trial included patients with dMMR/MSI-H advanced or recurrent EC who had progressed on or after no more than 2 prior lines of systemic chemotherapy (with ≥1 line of PBCT) and were then treated with dostarlimab. The Flatiron Health database (described in more detail in the following section) allows selection of real-world patients with inclusion/exclusion criteria matched as closely as possible to GARNET, creating an external real-world control arm and mimicking a 2-arm parallel design. Patient-level longitudinal data were available for both the GARNET trial cohort and the real-world cohort. The GARNET trial was performed in accordance with the principles of the Declaration of Helsinki, Good Clinical Practices, and all local laws.^20^

### Study Cohorts

The analysis data set for the GARNET arm consisted of the safety population from Cohort A1 of GARNET. The first subject-first visit took place on May 8, 2017, and the data cut-off was November 1, 2021, at which point enrollment in Cohort A1 was completed. The GARNET analysis data set (hereafter referred to as the GARNET trial cohort) included participants with dMMR/MSI-H advanced or recurrent EC who received any amount of dostarlimab, regardless of follow-up time at data cut-off (N = 153 patients). Patients in GARNET who had received anti-PD-(L)1/2 therapy as subsequent anticancer therapy following dostarlimab (n = 6) were excluded. Study locations for GARNET were the US, Canada, Denmark, Czech Republic, France, Italy, Poland, Spain, and the United Kingdom. The index date was defined as the day that patients received the first dose of dostarlimab.

To construct an external control arm, this study used the nationwide Flatiron Health EHR-derived de-identified database. The Flatiron Health database is a longitudinal database, comprising de-identified patient-level structured and unstructured data, curated via technology-enabled abstraction.^21^ During the study period, the de-identified data originated from approximately 280 cancer clinics (~800 sites of care). The Flatiron Health Endometrial Cancer Analytic Cohort is composed of patients who had an initial diagnosis of stage III or IV EC on or after January 1, 2013, or an initial diagnosis of stage I or II EC with subsequent locoregional or distant recurrence on or after January 1, 2013, and at least 2 documented clinical encounters on or after January 1, 2013.

Inclusion and exclusion criteria applied to the Flatiron Health Endometrial Cancer Cohort were matched as closely as possible to inclusion and exclusion criteria for GARNET. Patients were required to be at least 18 years of age at the time of advanced or recurrent EC diagnosis and to have received no more than 2 oncologist-defined, rule-based lines of systemic chemotherapy for advanced or recurrent EC, including at least 1 line of PBCT. They must also have received an additional oncologist-defined, rule-based line of therapy (LOT) following PBCT, which was classed as the index therapy (hormone monotherapy was allowed but did not count as an index therapy). They were also required to have an Eastern Cooperative Oncology Group (ECOG) performance status (PS) of 0-1 at index. Anti-PD-(L)1/2 treatments (eg, pembrolizumab) have received approval for the treatment of dMMR/MSI-H solid tumors (including EC) from the US and the EU regulatory authorities in the last 5 years.^22,23^ To minimize treatment differences between the US-based Flatiron cohort and the GARNET trial, which prohibited prior anti-PD-(L)1/2 therapy, patients who had received anti-PD-(L)1/2 therapy at any point were excluded. Patients diagnosed with a malignancy (excluding nonmelanoma skin cancer and carcinoma in situ cervix) that progressed or required active treatment no more than 2 years before index date, autoimmune disease, hepatitis B or C, or HIV were also excluded.

The index date for the real-world cohort was defined as the date of initiation of the post-platinum regimen (ie, 2L or third-line [3L] therapy) and must have occurred between January 1, 2013, and August 31, 2018.

The precise date was determined algorithmically; a patient’s potential eligible treatment pattern for up to 2 lines was identified, which could be (a) 1L PBCT only; (b) 1L and 2L PBCT; (c) 1L non-PBCT and 2L PBCT; or (d) 1L PBCT and 2L non-PBCT. For scenarios (a), (c), and (d), the index date was defined as the start date of the initiating LOT (index therapy) following the eligible treatment pattern. In scenario (b), where a patient had 2 PBCT LOTs, they could conceivably be assigned to scenario (a) or (b) at the point at which they were eligible for dostarlimab. These patients were randomly assigned to either (a) or (b) and the index date was determined accordingly. As patients enrolled in GARNET at the current point in their treatment journey and the real-world study was retrospective, this random assignment ensured that the patient journey on PBCT closely mirrored potential entry points into GARNET.

### Outcome Measures

The primary endpoint for comparison in this study was OS, defined as the interval between the index date and the date of death by any cause. Patients who did not die after the index date were censored at the latest date of follow-up (both cohorts) or data cut-off (GARNET trial cohort only).

The exploratory endpoint, TTD, was calculated as the duration from the start to the end of index therapy. Patients were censored if the patient died, was lost to follow-up, or was still on therapy. Patients in the real-world cohort were assumed to be either lost to follow-up or still on therapy if there was no evidence of confirmed structured activity during a period of 120 days after the last drug episode for the treatment of interest.

### Prognostic Factors

A targeted literature review was conducted in May 2020 to identify prognostic variables associated with survival in EC. Following consultation with a panel of physicians on the prognostic variables derived from literature review, the following prioritized prognostic factors were identified based on clinical relevance: race, age, ECOG PS, histology, International Federation of Gynecology and Obstetrics (FIGO) stage, body mass index, and grade of disease at initial EC diagnosis. MMR/MSI status was also identified as a prioritized prognostic factor, but these data were not consistently available in the Flatiron cohort. As highlighted earlier, however, there is conflicting evidence on the prognostic value of MMR/MSI status.^7–10^ After considering the relative data availability for each prognostic factor in the 2 cohorts, a propensity score model was built, including histology, grade of disease at initial EC diagnosis, ECOG PS, and number of lines of PBCT in the advanced or recurrent setting.

### Inverse Probability of Treatment Weighting and PSM

To control for the lack of randomization inherent to external control arm studies, treatment effectiveness was calculated using inverse probability of treatment weighting (IPTW).^24^ During IPTW adjustment, patients without prior PBCT treatment in the advanced or recurrent setting (n = 2) and patients with unknown histology (n = 2) were omitted from the GARNET trial cohort, as equivalent patients were not observed in the real-world cohort. Propensity scores for each patient in the real-world cohort were estimated based on the propensity score model (logistic regression), reflecting a patient’s predicted probability of being assigned to GARNET. IPTW was then performed using weights from estimated propensity scores, calculated so that resulting estimates referred to the average treatment effect. To avoid large weights, which could increase the variability of the estimated treatment effect, stabilized weights were used.

An alternative adjustment method, propensity score matching (PSM), was applied to the unadjusted data set and used as a sensitivity analysis for the stabilized IPTW–adjusted results. Matching was based on the greedy nearest neighborhood matching without replacement method, using the same propensity score model built for IPTW. A 1:2 matching (GARNET trial cohort : real-world cohort) was used to account for the imbalance in sample size between the 2 cohorts.

### Statistical Analysis

Following adjustment by PSM or stabilized IPTW, OS and TTD were compared between cohorts. Kaplan-Meier analysis was used to describe the distribution of OS and TTD by cohort. Weighted Kaplan-Meier curves were created following stabilized IPTW adjustment for the main analysis and after PSM adjustment for the sensitivity analysis. The adjusted hazard ratio (HR) was obtained for OS of dostarlimab compared with real-world treatments using a weighted Cox regression model following stabilized IPTW adjustment for the main analysis and following PSM adjustment for the sensitivity analysis. The proportional hazards assumption was checked graphically by means of log cumulative hazard plots for the covariate and by Schoenfeld residual plots. To assess if the proportional hazards assumption was met (non-constant hazards), an interaction between time and the study variable was also added to the Cox models as a time-dependent covariate. All data summaries and analyses were performed using SAS Version 9.4 or higher.

## RESULTS

### Baseline Characteristics

After applying all additional inclusion and exclusion criteria to the Flatiron Health Endometrial Cancer Analytic Cohort, the study included 185 patients diagnosed with advanced or recurrent EC from January 1, 2013, to August 31, 2018 (**Figure S1**). Patient baseline characteristics for the GARNET trial cohort (N = 147) and the real-world cohort (N = 185) before stabilized IPTW are summarized in **Table 1** (see **Table S1** for detailed FIGO stage breakdown). Patients in the GARNET trial cohort were similarly aged to those in the real-world cohort (mean age at index, 63.5 vs 64.2 years, respectively; **Table 1**), had a similar proportion of patients with advanced disease at diagnosis (58.5% vs 56.8% FIGO Stage III/IV, respectively; **Table 1**), and a similar proportion of patients with ECOG PS 1 (59.2% vs 53.5%, respectively). A slightly higher percentage of patients had endometrioid histology in the GARNET trial cohort compared with the real-world cohort (79.6% vs 57.3%, respectively; **Table 1**). A larger proportion of patients in the GARNET trial cohort were white compared with the real-world cohort (75.5% vs 61.1%, respectively; **Table 1**). Baseline and prognostic characteristics of the study populations after stabilized IPTW are also summarized in **Table 1**.

**Table 1.** Baseline Characteristics Before and After Stabilized IPTW Adjustment for the GARNET Trial Cohort Versus External Control Arm

**Baseline Characteristic**	**Before Stabilized IPTW Adjustment**		**After Stabilized IPTW Adjustment**			
	**GARNET Trial Cohort (N = 147), n (%)**	**Real-World Cohort (N = 185), n (%)**	GARNET Trial Cohort (N = 143*), %	**Real-World Cohort (N = 185), %**	**Standardized Difference, %**	***P* Value**
Age at index (y)			–	–	–	–
Mean (SD)	63.5 (8.8)	64.2 (9.7)				
Min, max	39.0, 85.0	30.0, 83.0	–	–	–	–
Age group	–	–				
<65			44.3	45.0	-0.02	.893
≥65			55.7	55.0	0.02	
Race						<.001
Asian	5 (3.4)	4 (2.2)	–	–	–	
Black/⁠African American	5 (3.4)	41 (22.2)	3.2	20.5	-0.55	
Other race	3 (2.0)	22 (11.9)	4.0	13.4	-0.34	
White	111 (75.5)	113 (61.1)	75.3	63.7	0.25	
Unknown	23 (15.6)	5 (2.7)	17.5	2.5	0.52	
Histology						.015
Endometrioid	117 (79.6)	106 (57.3)	80.4	68.3	0.28	
Non-endometrioid	28 (19.0)	79 (42.7)	19.6	31.7	-0.28	
Unknown	2 (1.4)	0 (0.0)	0	0	–	
FIGO stage at diagnosis					–	
Stage I	48 (32.7)	54 (29.2)	38.7 (I/II)	40.6 (I/II)	-0.04	.004
Stage II	13 (8.8)	12 (6.5)				
Stage III	55 (37.4)	29 (15.7)	61.3 (III/IV)	52.4 (III/IV)	0.18	
Stage IV	31 (21.1)	76 (41.1)				
Unknown	0 (0.0)	14 (7.6)	0.0	7.0		
Grade at initial diagnosis						
Grade 1	40 (27.2)	23 (12.4)	51.6	50.2	0.03	.911
Grade 2	57 (38.8)	47 (25.4)	27.4	26.7	0.01	
Grade 3	45 (30.6)	44 (23.8)	21.0	23.0	-0.05	
Unknown/not assessable	5 (3.4)	71 (38.4)				
ECOG PS						.868
0	60 (40.8)	86 (46.5)	45.1	44.2	0.02	
1	87 (59.2)	99 (53.5)	54.9	55.8	-0.02	
No. of prior PBCTs in advanced or recurrent setting						.686
0	2 (1.4)	0 (0.0)	0	0	0.05	
1	122 (83.0)	166 (89.7)	88.7	87.2	-0.05	
2+	23 (15.6)	19 (10.3)	11.3	12.8		

*Patients who did not receive any prior PBCTs in advanced or recurrent setting (n=2) and patients with unknown histology (n=2) were removed from the GARNET trial cohort as equivalent patients were not observed in the real-world cohort.

Abbreviations: ECOG PS, Eastern Cooperative Oncology Group performance status; FIGO, International Federation of Gynecology and Obstetrics; IPTW, inverse probability of treatment weighting; PBCT, platinum-based chemotherapy.

A total of 62 different index regimens were received by patients in the real-world cohort. Regimens received by at least 1% of patients are summarized in **Table 2**, with the most frequent being carboplatin plus paclitaxel (12.4%), pegylated liposomal doxorubicin monotherapy (10.3%), and bevacizumab (8.6%).

**Table 2.** Index Regimens Received by ≥1% of Patients in the Real-World Cohort

**Index Regimen**	**No. of Patients (N = 185), n (%)**
Anastrozole +	
Carboplatin + paclitaxel	2 (1.1)
	
Bevacizumab	16 (8.6)
Bevacizumab +	
Carboplatin	2 (1.1)
Carboplatin + paclitaxel	6 (3.2)
Doxorubicin, pegylated liposomal	8 (4.3)
Gemcitabine	2 (1.1)
Paclitaxel	3 (1.6)
Topotecan	2 (1.1)
	
Carboplatin +	
Docetaxel	13 (7)
Docetaxel + paclitaxel	2 (1.1)
Doxorubicin, pegylated liposomal	5 (2.7)
Gemcitabine	4 (2.2)
Paclitaxel	23 (12.4)
	
Cisplatin	2 (1.1)
Cisplatin +	
Doxorubicin	2 (1.1)
	
Clinical study drug	7 (3.8)
Docetaxel	3 (1.6)
Doxorubicin	3 (1.6)
Doxorubicin, pegylated liposomal	19 (10.3)
Gemcitabine	4 (2.2)
Paclitaxel	3 (1.6)
Temsirolimus	2 (1.1)
Topotecan	13 (7)

### Overall Survival

**Figure 1.**
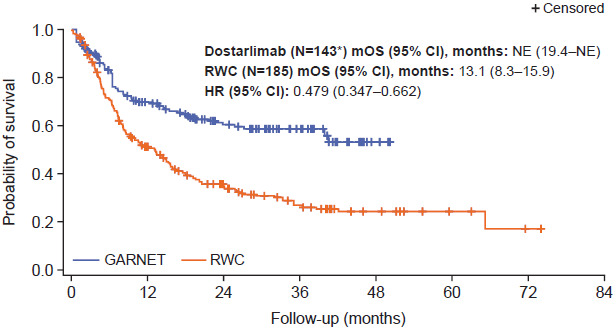
Overall Survival After Stabilized IPTW Adjustment in the GARNET Trial Cohort Versus Real-World Cohort *Patients who did not receive any prior PBCTs in advanced or recurrent setting (n=2) and patients with unknown histology (n = 2) were removed from the GARNET trial cohort as equivalent patients were not observed in the real-world cohort. Abbreviations: CI, confidence interval; HR, hazard ratio; IPTW, inverse probability of treatment weighting; mOS, median overall survival; NE, not estimable; PBCT, platinum-based chemotherapy; RWC, real-world cohort.

Prior to adjustment, median OS was not estimable (NE) (95% confidence interval [CI], 39.9 months–NE) for patients in the GARNET trial cohort compared with 11.1 months in the real-world cohort (95% CI, 8.1-15.2; **Figure S2**). When interaction between time and study was added to the Cox proportional hazards regression model, the interaction term was not statistically significant (*P* = .515), so it is reasonable to assume that the proportional hazards assumption was valid. This was supported by the Schoenfeld residual plot and log-cumulative hazard plot (**Figure S3**). Median OS after stabilized IPTW adjustment remained longer for patients treated with dostarlimab (N = 143) compared with patients receiving real-world non-anti-PD-(L)1/2 treatment regimens (NE [95% CI, 19.4-NE] vs 13.1 months [95% CI, 8.3-15.9], respectively; **Figure 1**). Analysis of stabilized IPTW-adjusted survival rates indicated that there was a larger proportion of patients in the GARNET trial cohort surviving compared with the real-world cohort at 6, 12, 18, 24, 36, and 48 months (**Table 3**). Accordingly, patients treated with dostarlimab had a 52% lower hazard of death compared with patients receiving real-world non-anti-PD-(L)1/2 treatments (HR after stabilized IPTW, 0.48 [95% CI, 0.35-0.66]; *P* < .001) (**Figure 1**). The interaction term was not statistically significant after stabilized IPTW adjustment (*P* = .146). However, the *P* value for the global Schoenfeld test was <.1 and the log-cumulative hazard plot showed some crossing of curves at the beginning, indicating a potential violation of the proportional hazards assumption and suggesting that the hazard ratio for the study variable should be interpreted with caution (**Figure S4**).

**Table 3.** Stabilized IPTW-Adjusted Overall Survival Rates

**Overall Survival Rate, % (95% CI)**	**GARNET Trial Cohort (N = 143)**	**Real-World Cohort (N = 185)**
6 months	83.1 (71.6-90.2)	70.9 (63.1-77.3)
12 months	69.8 (57.1-79.4)	51.2 (43.1-58.7)
18 months	64.7 (51.7-75.1)	39.7 (31.8-47.4)
24 months	60.4 (46.1-71.4)	34.8 (27.1-42.6)
36 months	58.7 (45.2-69.9)	27.0 (19.4-35.1)
48 months	53.2 (39.0-65.5)	24.3 (16.6-32.7)

Abbreviations: CI, confidence interval; IPTW, inverse probability of treatment weighting.

The sensitivity analysis based on PSM gave similar results, with the median OS being longer for patients treated with dostarlimab compared with patients receiving real-world non-anti-PD-(L)1/2 treatment regimens (NE [95% CI, 39.9-NE] vs 13.1 months [95% CI, 8.0–17.9], respectively) (**Figure S5**). Accordingly, patients treated with dostarlimab had a 54% lower hazard of death compared with patients receiving real-world non-anti-PD-(L)1/2 treatments (HR after PSM 0.455 [95% CI, 0.307-0.676]; *P* < .001) (**Figure S5**). The interaction term was not statistically significant after PSM adjustment (*P* = .687), and both the Schoenfeld residual plot and log-cumulative hazard plot suggested that the proportional hazards assumption is likely to hold (**Figure S6**).

### Time to Treatment Discontinuation

**Figure 2.**
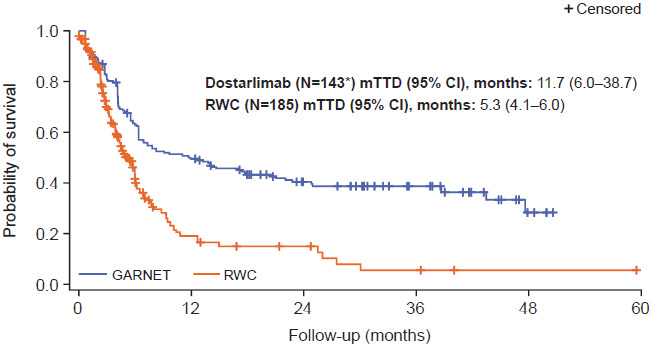
Time to Treatment Discontinuation After Stabilized IPTW Adjustment in the GARNET Trial Cohort Versus Real-World Cohort *Patients who did not receive any prior PBCTs in advanced or recurrent setting (n = 2) and patients with unknown histology (n = 2) were removed from the GARNET trial cohort as equivalent patients were not observed in the real-world cohort. Abbreviations: CI, confidence interval; IPTW, inverse probability of treatment weighting; mTTD, median time to treatment discontinuation; PBCT, platinum-based chemotherapy; RWC, real-world cohort.

Unadjusted TTD was longer for patients treated with dostarlimab compared with patients in the real-world cohort (9.9 months [95% CI, 6.1-17.0] vs 5.3 months [95% CI, 4.2-6.0], respectively; **Figure S7**). TTD following adjustment with stabilized IPTW was also longer for patients treated with dostarlimab than for patients treated with real-world regimens (11.7 months [95% CI, 6.0-38.7] vs 5.3 months [95% CI, 4.1-6.0]; **Figure 2**). The sensitivity analysis using PSM gave similar results, with patients receiving dostarlimab having a longer PSM-adjusted TTD compared with patients receiving real-world treatment (12.6 months [95% CI, 6.9-22.0] vs 5.7 months [95% CI, 3.9-6.2]; **Figure S8**).

## DISCUSSION

This study compared clinical outcomes between dostarlimab and currently utilized real-world non-anti-PD-(L)1/2 treatments in patients with advanced or recurrent EC who had progressed after no more than 2 prior lines of systemic chemotherapy (with ≥1 line of PBCT) in the US. An external control arm was constructed using patient-level data from the Flatiron Health database, which is derived from EHRs throughout the US. Patient-level data allows the best adjustment for selection bias resulting from lack of randomization in external control arm real-world evidence studies.^25^ The diversity of index treatment regimens in the real-world cohort highlights the lack of a standard of care for patients with advanced or recurrent EC in the 2L setting.

In this study, patients treated with dostarlimab had significantly longer OS than patients in the real-world cohort after adjusting for the lack of randomization by means of stabilized IPTW. This is consistent with previous analyses showing that dostarlimab-treated patients with advanced or recurrent EC had increased OS when compared with patients treated with the chemotherapy doxorubicin^26^ or with patients receiving real-world treatments in England.^27^ The longer OS seen with dostarlimab treatment compared with the external control arm could potentially be due to (1) the relatively high response rate seen in GARNET (objective response rate for the dMMR/MSI-H population: 45.5% [95% CI, 37.1%-54.0%])^28^ compared with the relatively low response rates observed with commonly used therapies,^3^ and (2) the durability of response seen in GARNET, where Kaplan-Meier analysis indicated a 90.9% chance of maintaining a response at 12 months, a 80.1% chance at 18 months,^20^ and an 83.7% chance of remaining in response at 24 months follow-up.^29^ Additionally, patients had a long TTD when treated with dostarlimab (9.9 months in the GARNET trial cohort), suggesting a favorable tolerability profile for dostarlimab, enabling patients to remain on treatment. This is supported by safety data from GARNET, which showed the majority of treatment-related adverse events to be mild to moderate (grade 1-2).^28^ Reasons for treatment discontinuation were not reported for the GARNET trial cohort in this study; however, in the GARNET safety population, treatment-related adverse events leading to treatment discontinuation were low, occurring in 13 of 153 patients (8%).^28^ Together with dostarlimab’s 6-week treatment schedule (after the first 4 doses, which are given once every 3 weeks), which is less intensive compared with many chemotherapy regimens, these data highlight the potential value of dostarlimab treatment to the patient beyond improved survival.

A sensitivity analysis was conducted for OS and TTD, using PSM as an alternative to stabilized IPTW. For both outcomes, PSM results supported those found using stabilized IPTW, with significantly longer OS and TTD seen in the GARNET trial cohort compared with the real-world cohort.

This study has several strengths and limitations. Despite the availability of patient-level data for both cohorts, there is always the possibility that differences in data collection or other unmeasured confounding variables could contribute to observed differences in treatment effect between the GARNET trial cohort and the real-world cohort. Data from EHRs, such as those included in the Flatiron Health database, are used for billing and clinical practice management and are not primarily designed for research purposes. In particular, the Flatiron data set analyzed in this study did not contain progression or response variables assessed according to Response Evaluation Criteria in Solid Tumors (RECIST) criteria (as in GARNET). Consequently, the ability to evaluate progression or response-related outcomes is limited. Further, ECOG PS was unknown for some patients in the Flatiron database; to align with GARNET eligibility criteria (requiring an ECOG PS of 0 or 1), patients with unknown ECOG PS were excluded from the real-world cohort. However, as missing ECOG PS data are likely to be due to administrative errors, we would not expect this to bias composition of the real-world cohort or impact the study results. The LOT defined by Flatiron may also not reflect the exact treatment path. Comparability of certain variables, such as prior surgery and radiation, is limited by underreporting, so these were consequently not used in propensity score models for this analysis. There were also fewer non-white patients in the GARNET trial cohort compared with the external control arm; this may reflect the international nature of the GARNET trial in contrast to the US-based Flatiron Health database as well as a widespread issue with inclusion of racial and ethnic minorities in cancer clinical trials.^30^ Nonetheless, external control arms are recognized by regulatory and HTA authorities,^18,19^ with evidence from such studies supporting drug regulatory applications and HTA submissions.^31^ In addition, comparisons with a Flatiron-based external control arm have previously been used to support the expansion of indications for other oncology drugs in the US,^32^ as well as HTA submissions,^33^ supporting its robustness as a real-world control cohort in the current study. An additional limitation is the potential violation of the proportional hazard assumption in the Cox regression model of stabilized IPTW-adjusted OS, suggesting the hazard ratio should be interpreted with caution. However, the HRs for both unadjusted and PSM-adjusted OS, where the proportional hazard assumption was likely to hold, support the HR for stabilized IPTW-adjusted OS, suggesting that the potential violation did not have a substantial impact on the study results.

While the time period considered for the start of index therapy in the real-world cohort (January 1, 2013–August 31, 2018) overlaps with the enrollment period for GARNET (May 8, 2017–November 1, 2021 [data cut-off, enrollment ongoing]), they do not align completely as the period for the external control arm was expanded to generate a sufficient sample size for analysis. The majority of chemotherapies (eg, taxanes, anthracyclines, PBCT) currently used to treat advanced or recurrent EC were already available and/or recommended during the indexing period for the real-world cohort, with bevacizumab being the only new commonly-used therapy.^34–36^ The differences in enrollment period length may affect censoring rates due to a longer follow-up time being available for the real-world cohort versus GARNET. Additionally, the longer time period for the real-world cohort may have contributed to MMR/MSI status not being consistently reported in the Flatiron database. While the Society for Gynecologic Oncology has recommended MMR/MSI testing in the US for EC since 2014, this guidance has not been consistently applied.^37^ As highlighted previously, there is no consistent evidence on the prognostic value of MMR/MSI status in advanced or recurrent EC, with marked interstudy heterogeneity.^7–10,38^ Recent data from the KEYNOTE-775 study showed a lower OS for patients with dMMR EC than patients with MMRp EC receiving chemotherapy.^39^ While this is an evolving area of understanding, overall, these data suggest that differences in MMR/MSI status between the GARNET and Flatiron cohorts should not contribute substantially to the difference in survival outcomes observed in this analysis.

Both stabilized IPTW and PSM models were adjusted for endometrioid versus non-endometrioid histology. More specific histological subtypes (eg, serous) within the non-endometrioid category could not be included in the statistical models as the sample size for each individual non-endometrioid histological subtype was insufficient and the reporting of subtypes differed across the cohorts. Discrepancies in the number of patients with different non-endometrioid histological subtypes between the GARNET trial cohort and the real-world cohort is a potential confounding variable for this analysis.

Finally, this analysis only evaluated non-anti-PD-(L)1/2 treatments. Another anti-PD-1 antibody, pembrolizumab, was approved in the US for use in dMMR/MSI-H solid tumors (including EC) towards the end of the Flatiron enrollment period (May 2017).^40^ However, previous treatment with anti-PD-(L)1/2 therapy was an exclusion criterion for GARNET. If included, patients treated with pembrolizumab would represent a very small subpopulation in the real-world cohort with a short follow-up time and a disparate survival profile compared with patients receiving chemotherapy, which may affect the validity of the analysis.

## CONCLUSION

By comparing the GARNET trial cohort to a real-world external control arm from a multicenter oncology database in the US, this study found that patients with advanced or recurrent EC receiving dostarlimab had significantly better survival outcomes than patients receiving currently available non-anti-PD-(L)1/2 treatments. This finding still held true after adjusting for potential imbalances in baseline and prognostic factors and after sensitivity analyses using different statistical techniques. Overall, these findings suggest that dostarlimab monotherapy could bring additional clinical benefits to patients with advanced or recurrent EC who progress on or after PBCT. This could help inform treatment decisions in clinical practice and could also inform other healthcare decision makers when considering patient access to more efficacious treatments for patients with advanced or recurrent dMMR/MSI-H EC.

### Data Availability Statement

The data that support the findings of this study have been originated by Flatiron Health, Inc. These de-identified data may be made available upon request and are subject to a license agreement with Flatiron Health; interested researchers should contact DataAccess@flatiron.com to determine licensing terms. GSK makes available anonymized individual participant data and associated documents from interventional clinical studies that evaluate medicines, upon approval of proposals submitted to https://www.gsk-studyregister.com/en/. To access data for other types of GSK sponsored research, for study documents without patient-level data and for clinical studies not listed, please submit an enquiry via the website.

## Supplementary Material

Online Supplementary Material
