# Supplementary material for: Outcomes for Dostarlimab and Real-World Treatments in Post-platinum Patients With Advanced/Recurrent Endometrial Cancer: The GARNET Trial Versus a US Electronic Health Record-Based External Control Arm
Source: J Health Econ Outcomes Res. 2023 Sep 8;10(2):53–61. doi: 10.36469/001c.77484 (PMC10493167; doi:10.36469/001c.77484)
Supplement: Online Supplementary Material [file jheor_2023_10_2_77484_180313.pdf]

Journal of Health Economics and Outcomes Research

Online Supplementary Material

Outcomes for Dostarlimab and Real-World Treatments in Post-platinum Patients With Advanced or Recurrent Endometrial Cancer: The GARNET Trial Versus a US Electronic Health Record–based External Control Arm. *JHEOR*. 2023;10(2):?53-61. doi:10.36469/jheor.2023.77484

Table S1: Detailed FIGO Stage at Diagnosis Before Stabilized-IPTW Adjustment

Figure S1: Selection of Patients from the Flatiron Health Endometrial Cancer Analytic Cohort

Figure S2: Unadjusted Overall Survival for the GARNET Trial Cohort versus Real-World Cohort

Figure S3: (A) Log Cumulative Hazard Plot and (B) Schoenfeld Residual Plot for Unadjusted Overall Survival for GARNET Trial Cohort versus Real-World Cohort

Figure S4: (A) Log Cumulative Hazard Plot and (B) Schoenfeld Residual Plot for Stabilized IPTW-Adjusted Overall Survival for GARNET Trial Cohort versus Real-World Cohort

Figure S5: Overall Survival After PSM for the GARNET Trial Cohort versus Real-World Cohort

Figure S6: (A) Log Cumulative Hazard Plot and (B) Schoenfeld Residual Plot for PSM-Adjusted Overall Survival for GARNET Trial Cohort versus Real-World Cohort

Figure S7: Unadjusted Time to Treatment Discontinuation for GARNET Trial Cohort versus Real-World Cohort

Figure S8: Time to Treatment Discontinuation After PSM for GARNET Trial Cohort versus Real-World Cohort

This supplementary material has been provided by the authors to give readers additional information about their work.

This is an open-access article distributed under the terms of the Creative Commons Attribution 4.0 International License (CCBY-4.0). View this license's legal deed at http://creativecommons.org/licenses/by/4.0 and legal code at http://creativecommons.org/licenses/by/4.0/legalcode for more information.

Table S1. Detailed FIGO Stage at Diagnosis Before Stabilized-IPTW Adjustment

FIGO Stage at EC Diagnosis	GARNET Trial Cohort (N = 147), n (%)	Real-World Cohort (N = 185), n (%)
Stage I	3 (2.0)	2 (1.1)
Stage IA	23 (15.6)	27 (14.6)
Stage IB	22 (15.0)	23 (12.4)
Stage IC	0 (0.0)	2 (1.1)
Stage II	13 (8.8)	12 (6.5)
Stage III	4 (2.7)	1 (0.5)
Stage IIIA	12 (8.2)	6 (3.2)
Stage IIIB	5 (3.4)	4 (2.2)
Stage IIIC	9 (6.1)	18 (9.7)
Stage IIIC1	10 (6.8)	0 (0.0)
Stage IIIC2	15 (10.2)	0 (0.0)
Stage IV	13 (8.8)	1 (0.5)
Stage IVA	1 (0.7)	2 (1.1)
Stage IVB	17 (11.6)	73 (39.5)
Unknown	0 (0.0)	14 (7.6)

Abbreviations: EC, endometrial cancer; FIGO, International Federation of Gynecology and Obstetrics; IPTW, inverse probability of treatment weighting.

Figure S1. Selection of Patients from the Flatiron Health Endometrial Cancer Analytic Cohort

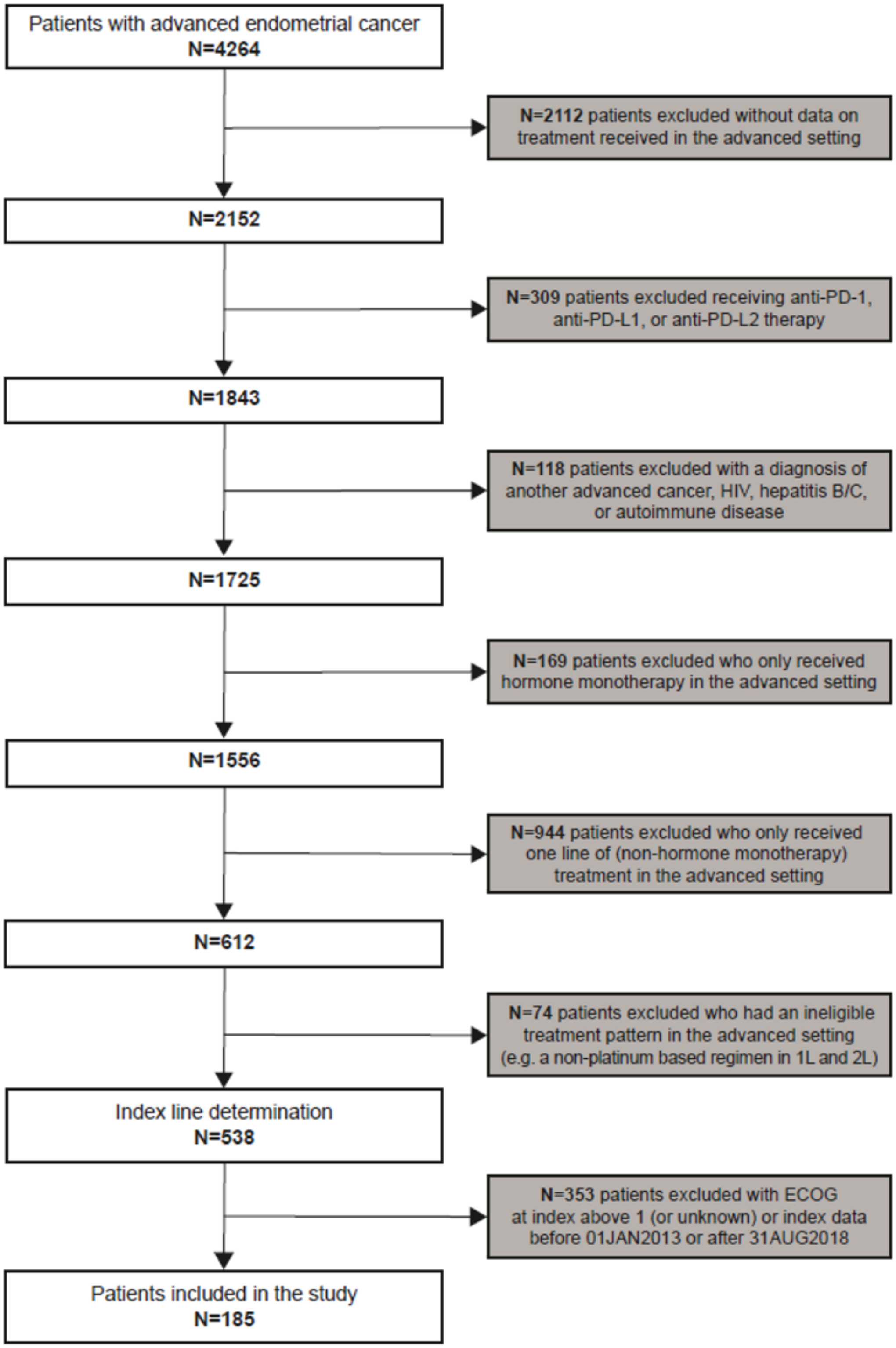

Abbreviations: 1/2L, first-/second-line; ECOG, Eastern Cooperative Oncology Group; PD-(L)1/2, programmed death-(ligand)1/2.

Figure S2. Unadjusted Overall Survival for the GARNET Trial Cohort Versus Real-World Cohort

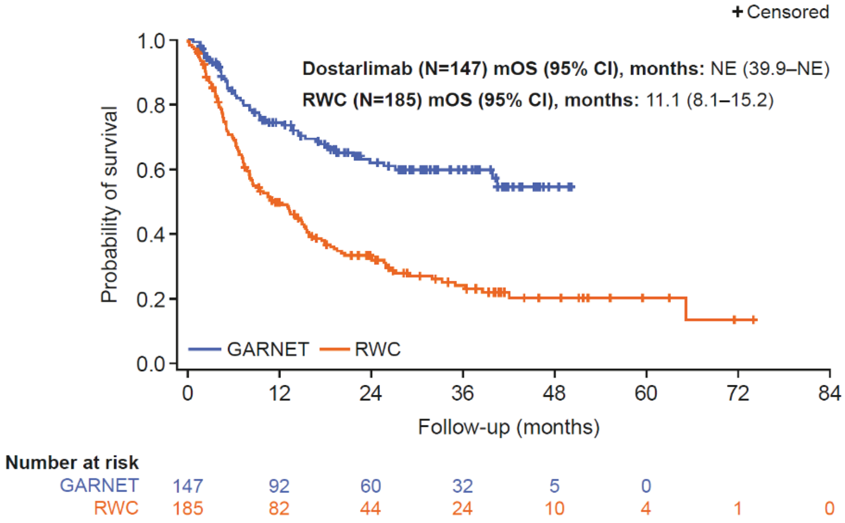

Abbreviations: CI, confidence interval; mOS, median overall survival; NE, not estimable; RWC, real-world cohort.

Figure S3. (A) Log Cumulative Hazard Plot and **(B)** Schoenfeld Residual Plot for Unadjusted Overall Survival for GARNET Trial Cohort Versus Real-World Cohort

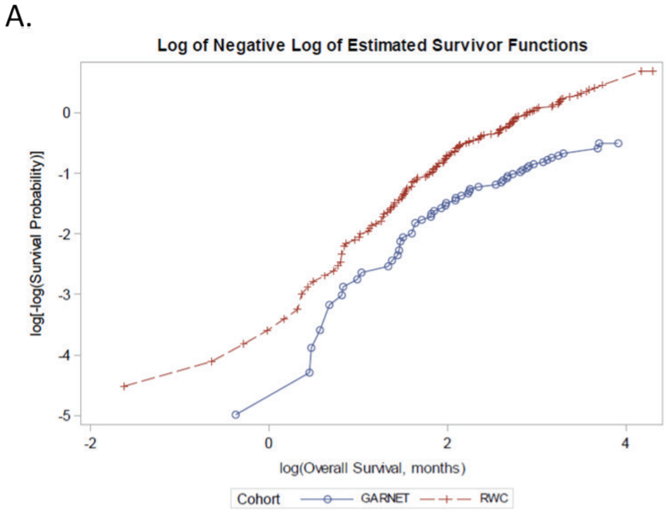

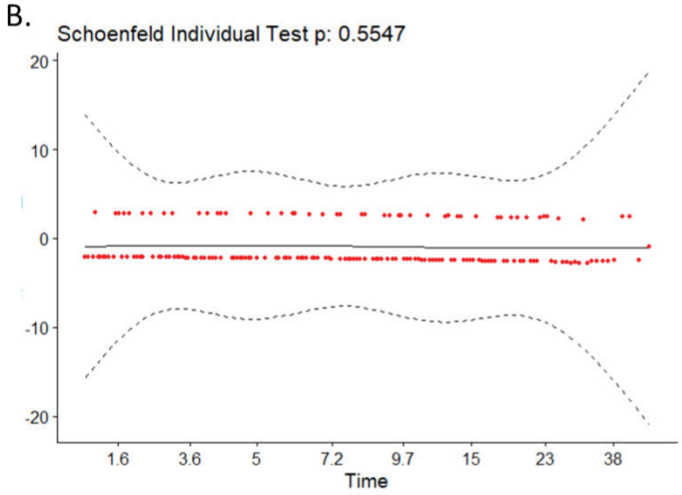

Abbreviation: RWC, real-world cohort.

Figure S4. (A) Log Cumulative Hazard Plot and (**B**) Schoenfeld Residual Plot for Stabilized-IPTW-Adjusted Overall Survival for GARNET Trial Cohort Versus Real-World Cohort

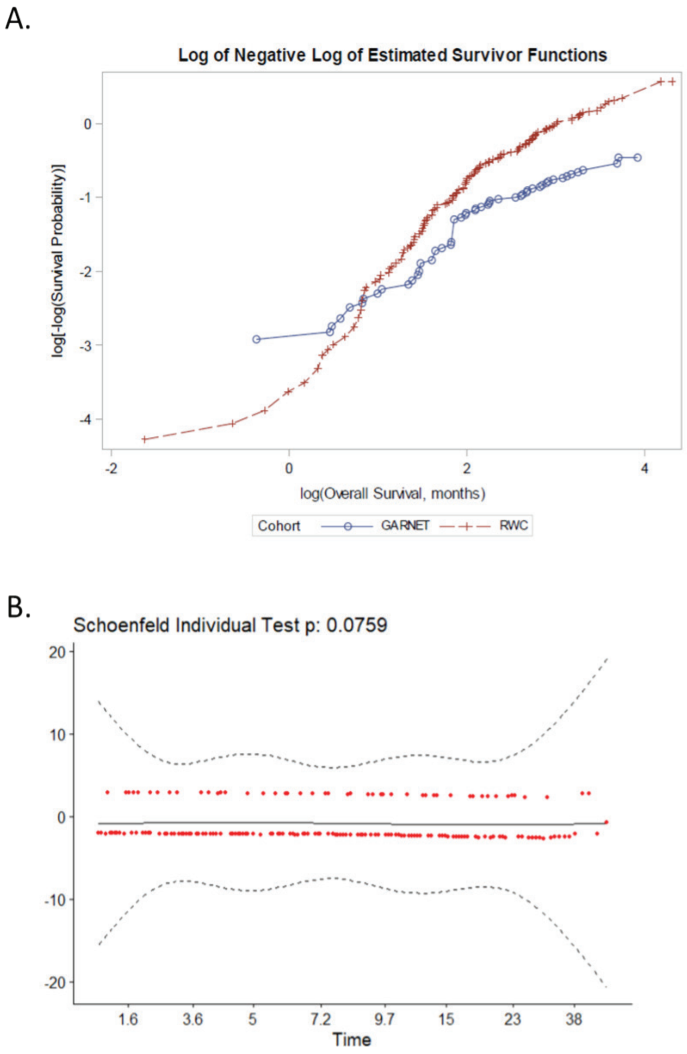

Abbreviations: IPTW, inverse probability of treatment weighting; RWC, real-world cohort.

Figure S5. Overall Survival After PSM for the GARNET Trial Cohort Versus Real-World Cohort

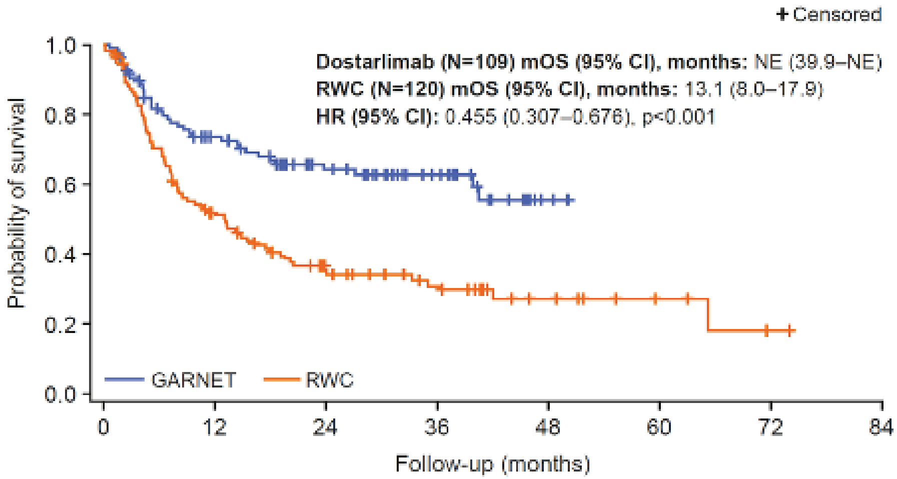

Abbreviations: CI, confidence interval; HR, hazard ratio; mOS, median overall survival; NE, not estimable; PSM, propensity score matching; RWC, real-world cohort.

Figure S6. (A) Log Cumulative Hazard Plot and (**B**) Schoenfeld Residual Plot for PSM-Adjusted Overall Survival for GARNET Trial Cohort Versus Real-World Cohort

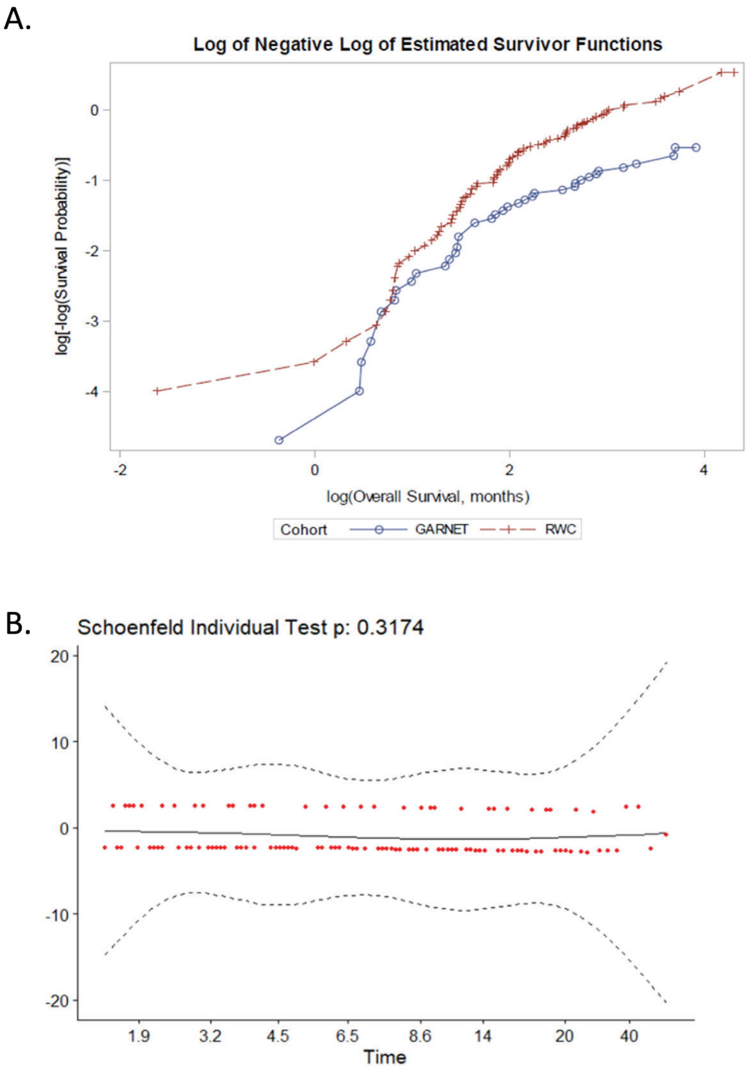

Abbreviations: PSM, propensity score matching; RWC, real-world cohort.

Figure S7. Unadjusted Time to Treatment Discontinuation for GARNET Trial Cohort Versus Real-World Cohort

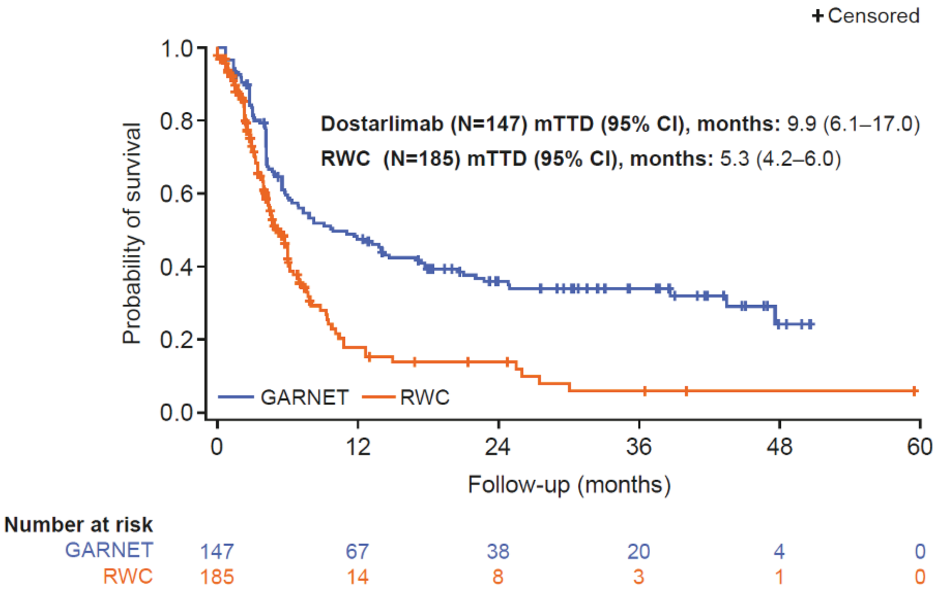

Abbreviations: CI, confidence interval; mTTD, median time to treatment discontinuation; NE, not estimable; RWC, real-world cohort.

Figure S8. Time to Treatment Discontinuation After PSM for GARNET Trial Cohort Versus Real-World Cohort

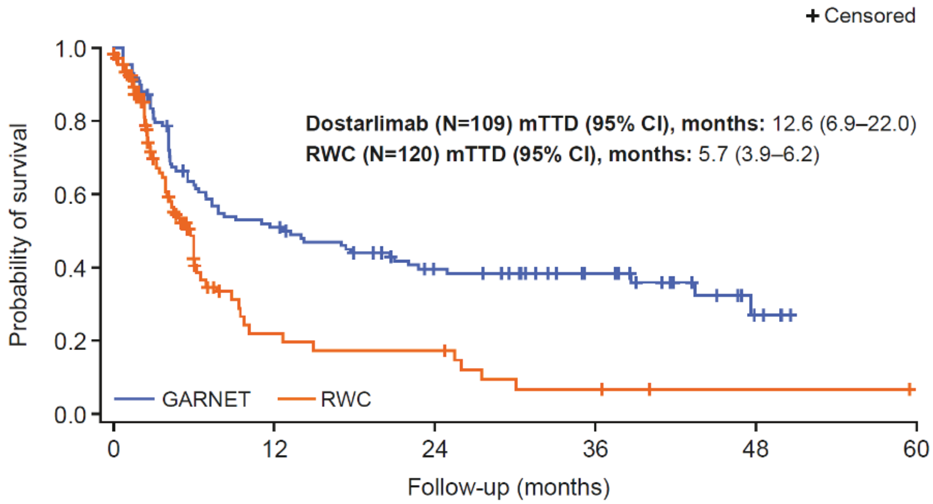

Abbreviations: CI, confidence interval; mTTD, median time to treatment discontinuation; NE, not estimable; PSM, propensity score matching; RWC, real-world cohort.
